# Modification and application of highly active alkaline pectin lyase

**DOI:** 10.1186/s13568-022-01472-0

**Published:** 2022-10-09

**Authors:** Pi-Wu Li, Jun Ma, Xiao-Feng Wei, Zi-Yang Zhang, Rui-Ming Wang, Jing Xiao, Jun-Qing Wang

**Affiliations:** 1 State Key Laboratory of Biobased Material and Green Papermaking (LBMP), Qilu University of Technology, Jinan, 250353 Shandong Republic of China; 2 Key Laboratory of Shandong Microbial Engineering, Qilu University of Technology (Shandong Academy of Sciences), Jinan, 250353 Shandong Republic of China

**Keywords:** Alkaline pectin lyase, Fragment replacement, Enzymatic activity, Molecular dynamics simulation

## Abstract

Alkaline pectate lyase has developmental prospects in the textile, pulp, paper, and food industries. In this study, we selected BacPelA, the pectin lyase with the highest expression activity from *Bacillus clausii*, modified and expressed in *Escherichia coli* BL21(DE3). Through fragment replacement, the catalytic activity of the enzyme was significantly improved. The optimum pH and temperature of the modified pectin lyase (PGLA-rep4) were 11.0 and 70 °C, respectively. It also exhibited a superior ability to cleave methylated pectin. The enzyme activity of PGLA-rep4, measured at 235 nm with 0.2% apple pectin as the substrate, was 554.0 U/mL, and the specific enzyme activity after purification using a nickel column was 822.9 U/mg. After approximately 20 ns of molecular dynamics simulation, the structure of the pectin lyase PGLA-rep4 tended to be stable. The root mean square fluctuation (RMSF) values at the key catalytically active site, LYS168, were higher than those of the wildtype PGLA. In addition, PGLA-rep4 was relatively stable in the presence of metal ions. PGLA-rep4 has good enzymatic properties and activities and maintains a high pH and temperature. This study provides a successful strategy for enhancing the catalytic activity of PGLA-rep4, making it the ultimate candidate for degumming and various uses in the pulp, paper, and textile industries.

**Supplementary Information:**

The online version contains supplementary material available at 10.1186/s13568-022-01472-0.

## Introduction

Pectin is an important, complexly structured anionic heteropolysaccharide. It is a critical component of the primary cell wall and the middle glue of plants (Hadi et al. 2021). The molecular structure of pectin can be roughly divided into three types: rhamnogalacturonan I (RG-I), rhamnogalacturonan II (RG-II), and homogalacturonan (HG) (Lin et al. 2021). At present, based on the pectin molecular domain-connection method, it is believed that the galacturonic acid-containing unit is the “smooth region,” while the part containing rhamnogalacturonan I and II is called the “hairy region” (Adetunji et al. 2017).

Pectin has diverse biological activities; it can activate the complement proteins in vitro, enhance neutrophil, macrophage, and lymphocyte activities, and has immunomodulatory effects (Zaitseva et al. 2020), which include enhancing T cell function and inhibiting myeloid-derived cell activity (Jeon et al. 2011). Additionally, pectin inhibits cancer cell proliferation (Delphi and Sepehri 2016; Fan et al. 2010; Guan et al. 2018), scavenges free radicals in organisms (Chen et al. 2016; Ogutu et al. 2016; Liu et al. 2016a), accelerates blood glucose metabolism (Jiao et al. 2014; Liu et al. 2016b), promotes the production of short-chain fatty acids such as acetic, propionic, and butyric acids, and maintains the health of the intestinal tract (Ferreira-Lazarte et al. 2018). Therefore, with the developmental strides of the food industry in recent years, and the consequent promotion of additives, pectin has become invaluable for its use as an emulsifier, thickener, stabilizer, and gelling agent and has been widely used in the food industry (Wang et al. 2021; Sun et al. 2020; Khubber et al. 2021; Li et al. 2019).

Complete pectin catabolism requires synergy between multiple pectinases with different substrate specificities (Kamijo et al. 2018). Therefore, pectinase is a general term for a class of enzymes involved in pectin degradation in plant cell tissues (Saharan and Sharma 2019). Based on substrates and mechanism of action, pectinase is classified into the following sub-types: protopectinase, pectinesterase, and depolymerase (Kashyap et al. 2001). The latter can be further divided into hydrolase and lyase. Hydrolase breaks the α-1,4 glycosidic bond of the pectin polymer main chain, while lyase breaks the main chain by trans-elimination, creating an unsaturated bond at the non-reducing end (Fu et al. 2008). Pectin lyase is an important industrial enzyme with commercial value, mainly derived from microorganisms: 50% from fungi and yeast and 35% from bacteria. Approximately 15% of pectin lyases are derived from animals or plants (Demir et al. 2014). The main bacterial species that contain pectate lyase include *Bacillus, Erwinia, Penicillium*, and *Aspergillus* (El-Rahim et al. 2020; Ribeiro et al. 2010; Yang et al. 2020). Pectinases can be classified as acid or alkaline based on their optimal pH range. Acid pectinase is mainly used for fruit juice extraction and processing (Cerreti et al. 2017; Sharma et al. 2017; Bhattacharyya et al. 2021). “Alkaline pectinase” generally refers to polygalacturonic acid (PGA) lyase. It has a more extensive application range, being used in the dyeing and processing of cotton and linen textiles, degumming of plant fibers, vegetable oil extraction, and the pulp and paper industry (Mudnoor and Chakraborty 2020; Cui et al. 2019; Hadj-Taieb et al. 2006; Huber et al. 2001; Camarero et al. 2003; Gil et al. 2009).

The potential market of alkaline pectin lyase is increasing due to its wide variety of applications. Nevertheless, its industrial applications are limited. Under some of the required processing conditions, its stability and enzyme activity is decreased, and its efficiency in degumming ramie, cotton textiles, and pulp processing is not ideal (Xu et al. 2021; Zheng et al. 2020; Cheng et al. 2019; Chiliveri and Linga 2014). Therefore, an alkaline pectin lyase with increased stability, heat resistance, and enzymatic activity is imperative to industrial processing.

In this study, we selected an alkaline pectin lyase, identified its physicochemical properties, and improved its enzymatic activity through a semi-rational design method to maintain its comprehensive properties, which can be applied to various research fields.

## Materials and methods

### Strains, plasmids, and materials

The pET28a(+) vector (GenScript Biotechnology Co., Ltd, China), pretreated with *Nco*I and *Xho*I, was used to clone the alkaline pectin lyase (*BacPelA*) gene (NCBI accession no. KR819891.1) from *Bacillus clausii* synthesized by GenScript Bioengineering Co., Ltd, China. *Escherichia coli* BL21 (DE3) (laboratory storaged) was the gene expression analysis host strain. TB culture medium was purchased from Beijing Coolebo Technology Co., Ltd, China. Apple and citrus pectin and polygalacturonic acid (PGA) were purchased from Sigma-Aldrich (St. Louis, MO, USA). All restriction enzymes were obtained from Thermo Fisher Scientific (China) Co., Ltd. Medium preparation reagents, agar and yeast extract powder, and peptone were obtained from Beijing Aoboxing Biotechnology Co., Ltd, China. Kanamycin, ampicillin, and isopropyl-β-d-thiogalactoside (IPTG) were purchased from Qingdao Sangon Biotechnology Co. Ltd, China. The chemicals used to prepare the buffers and other reagents were of reagent grade.

### Gene cloning and expression plasmid construction of PGLA

The NCBI BLAST program was used to search for the nucleotide sequence of *BacPelA* (Zhou et al. 2017a). The SignalP5.0 server (http://www.cbs.dtu.dk/services/SignalP/) was used to predict the signal peptide, which was removed. The *BacPelA* encoded DNA fragment comprised 304 amino acids (AA), with a total of 912 base pairs. The fragment’s two ends were modified with *Nco*I and *Xho*I, subjected to codon optimization to obtain the gene PGLA (accession no. OP355468), and were then ligated to the pretreated pET28a(+) vector, creating the ligation product pET28a(+)-*PGLA* (Additional file 1: Fig. S1). This recombinant plasmid was PGLA and then was transformed into *E.coli* BL21(DE3) competent cells, spread on Luria-Bertani (LB) agar (yeast dip powder, peptone, and NaCl; 5, 10, and 10 g/L, respectively) plates containing 50.0 µg/mL kanamycin, and incubated overnight at 37 °C. The cultured colonies were collected using a sterile pipette tip and transferred to tubes containing 1 mL of LB solution. These were shaken and incubated at 37 °C for 14 h. Thereafter, 1 mL of 50% sterilized glycerol was added to each tube, and the components were mixed and stored at – 80 °C.

### Modification of gene sequence of mutated alkaline pectin lyase with high enzyme activity

Sukhumsiirchart et al. (2009) studied pectin lyase Pel SWU (accession no. AB428424) from *Bacillus* sp. RN1, which has superior heat resistance compared to other similar enzymes. The NCBI program provided the nucleotide sequence of *Pel SWU*. The SignalP5.0 server predicted the signal peptides, which were subsequently removed. The 5′- and 3′-ends of the fragment were modified using *Nco*I and *Xho*I, respectively, and then ligated into the pretreated pET22b vector (GenScript Biotechnology Co., Ltd, China), creating the ligation product pET22b*-Pel*.

The AA sequences of *PGLA* and *Pel* were compared using ESPript 3.0 (https://espript.ibcp.fr/ESPript/ESPript/index.php). Four DNA fragments had a low sequence similarity at the N-terminus; this part was replaced. All the oligonucleotides used in the fragment replacement procedure are listed in Additional file 1: Table S1. The first replaced 5′ fragment was short. Thus, we used the PGLA1-F and PGLA1-R primer pairs to clone the entire recombinant plasmid (pET28a(+)-*PGLA-rep1*), via inverse PCR, with the pectin lyase pET28a(+)-*PGLA* DNA as the template. The second, third, and fourth N-terminal DNA fragments were then replaced by seamless cloning.

The replacement gene vector fragment was obtained by PCR amplification of the pectin lyase pET28a(+)-*PGLA* DNA template, with the primer pairs PGLA2-F, PGLA34-F, PGLA23-R, and PGLA4-R. The PCR amplification conditions are as follows: one reaction cycle at 95 °C for 3 min; pre-denaturation at 95 °C for 15 s, denaturation at 60 °C for 15 s, and extension at 72 °C for 3.2 min; 30 final extension cycles at 72 °C for 5 min; and storage at 4 °C.

We obtained the replacement gene fragment via PCR amplification using the primer pairs rep23-F, rep4-F, rep2-R, rep3-R, and rep4-R and the DNA template of pectin lyase pET22b-*Pel*. PCR amplification conditions: one cycle of reaction at 95 °C for 3 min; 30 cycles of pre-denaturation at 95 °C for 15 s, denaturation at 60 °C for 15 s, and extension at 72 °C for 20 s; a final extension at 72 °C for 5 min; and storage at 4 °C.

The above three pairs of DNA fragments were subjected to sodium dodecyl sulfate-polyacrylamide gel electrophoresis (SDS-PAGE). Thereafter, gel recovery was carried out with a product purification kit (Nanjing Novizan Biotechnology Co., Ltd, China), and the concentration of DNA fragments was measured with an MD2000 ultra-micro spectrophotometer (Shanghai Meixi Instrument Co., Ltd, China). The replacement gene fragment and vector fragment were ligated using the seamless cloning kit C112 (Nanjing Novizan Biotechnology Co., Ltd, China) to obtain the recombinant plasmids pET28a(+)*-PGLA-rep2*, pET28a(+)*-PGLA-rep3* and pET28a(+)-*PGLA-rep4*. The recombinant plasmids were transformed into *E. coli* BL21(DE3) competent cells, spread on LB agar plates and incubated overnight at 37 °C. The cultured colonies were collected using a sterile pipette tip and transferred to a tube containing 1 mL of LB solution. The tubes were shaken and incubated at 37 °C for 14 h. Thereafter, 1 mL of 50% sterilized glycerol was added to each tube, mixed, and stored at – 80 °C.

### Culture conditions for expression of alkaline pectin lyase in *E. coli*

Approximately 100 µL of frozen (– 80 °C) glycerol bacteria was added to 50 mL of LB medium containing 50 µg/mL kanamycin and cultured overnight at 37 °C to collect exponential phase cells. 300 uL seed medium containing exponential growth cells was inoculated into 50 mL TB medium containing 50 ug/mL kanamycin. The content of these flasks was cultured at 37 °C and 200 r/min. When the optical density at 600 nm (OD_600_) reached 0.6–0.8, isopropyl-β-d-thiogalactoside (IPTG) was added at a final concentration of 0.5 mM. Consequently, the protein was expressed in shaking flasks (200 r/min) for 24 h at 25 °C.

### Determination method and operation steps of enzyme activity

The activity of alkaline pectin lyase was assayed by measuring the increase in unsaturated bonds at 235 nm (A235 method). The fermentation broth (30 mL) was centrifuged to remove the supernatant, resuspended the pellet in 8 mL of phosphate-buffered saline (pH 7.4), and sonicated for 20 min. After centrifugation, the supernatant was collected to obtain the enzyme solution and stored at 4 °C. The enzyme activity assay was performed as described by Zhou et al. (2017a) with some modifications. We added 190 mL of glycine-NaOH (Gly-NaOH) buffer (pH 11.0), containing 0.2% pectin substrate and 100 µL of appropriately diluted enzyme solution, to a 25 mL colorimetric tube. The contents were mixed and allowed to react at 70 °C for 10 min. The reaction was terminated by adding 3 mL of 30.0 mM H_3_PO_4_. We used an V-5600 (PC) UV–Vis spectrophotometer (Shanghai Youke Instrument Co., Ltd, China) to measure the absorbance of the unsaturated product at 235 nm. One enzymatic activity unit is defined as the amount of enzyme required to cleave PGA to produce an equivalent of 1 µmol unsaturated oligogalacturonic acid per min. The molar absorption coefficient of unsaturated polygalacturonic acid at 235 nm was 4600 L/mol/cm. All enzyme activity measurements were performed in triplicate.

### Effects of pH, temperature and metal ions on enzyme activity and stability

To determine the optimum pH, we measured the absorbance of the enzyme solution at pH 8.5–11.5 with 50 mM Gly-NaOH buffer containing 0.2% apple pectin at 55 °C for 10 min. The optimal temperature was determined by measuring the absorbance of the enzyme solution (pH 11.0) at 55–85 °C (intervals of 5 °C) for 10 min. The pH stability was determined by the residual enzyme activity after 7 h incubation in Na_2_HPO_4_-citrate buffer (pH 4.0–7.0), 50 mM Tris-HCl buffer (pH 8.0), and Gly-NaOH buffer (pH 9.0–12.0) at 50 °C. The stability of pectin lyase was measured every hour for 5 h based on the residual enzyme activity after incubating in Gly-NaOH buffer (pH 11.0 and 12.0) at 25 °C. The thermal stability of pectin lyase was measured every hour for 5 h by testing the residual enzyme activity after incubating the enzyme solution at 60 and 70 °C. All experiments were repeated three times.

We incubated the enzyme, at room temperature, for 60 min, in a solution containing 1 mM of one of the following metal ions: K^+^, Ca^2+^, Na^+^, Mg^2+^, Cu^2+^, Mn^2+^, Fe^2+^, Zn^2+^, Fe^3+^, and Ni^+^. We then measured the relative enzyme activity under standard reaction conditions (pH 11.0, 70 °C) to determine the effect of metal ions on enzyme activity. We performed a control assay in the absence of metal ions to determine purified enzyme activity.

### Substrate specificity, molecular dynamics simulation, and kinetic parameter calculation

We determined substrate specificity by measuring the residual enzymatic activity of purified PGLA-rep4 on different substrates, including apple and citrus pectin and PGA, at a concentration of 0.2% under standard conditions (pH 11.0, 70 °C). Using the Swiss-model website (https://swissmodel.expasy.org/), we performed a sequence alignment on the five pectin lyases before and after the fragment was replaced. Thereafter, the Modeller 10.2 software (University of California San Francisco, San Francisco, CA, USA; http://salilab.org/modeller) was used to perform three-dimensional (3D) modeling. Molecular dynamics (MD) simulations of PGLA, PGLA-rep1, PGLA-rep2, PGLA-rep3, and PGLA-rep4 were performed using Gromacs 4.5 package (Royal Institute of Technology, Stockholm, and Uppsala University, Uppsala, Sweden; http://www.gromacs.org/), with the GROMOS 96 forcefield, and a simple point charge (SPC) water model. The protein was placed in a square box, with the edge of the box no closer than 1.5 nm to the protein, and 15,000 water molecules were added to the solvate proteins. After adding two Na^+^, the net charge of the system was zero, reaching an equilibrium state. Subsequently, to minimize the energy of the system, 1500 steps of steep descent and 2000 conjugate gradients were performed. Molecular dynamics simulations were performed at a constant temperature and pressure for 20 ns, with each step comprising 0.02 ps. The B-factor value of the amino acid residue was generated after MD simulation of the three-dimensional structure of the protein, that is, the atomic displacement parameter. The K_m_ and V_max_ enzyme values were calculated using nonlinear regression. All data were expressed as the average of three experiments.

## Results

### Construction, expression, and purification of recombinant alkaline pectin lyase with high enzyme activity

**Fig. 1:**
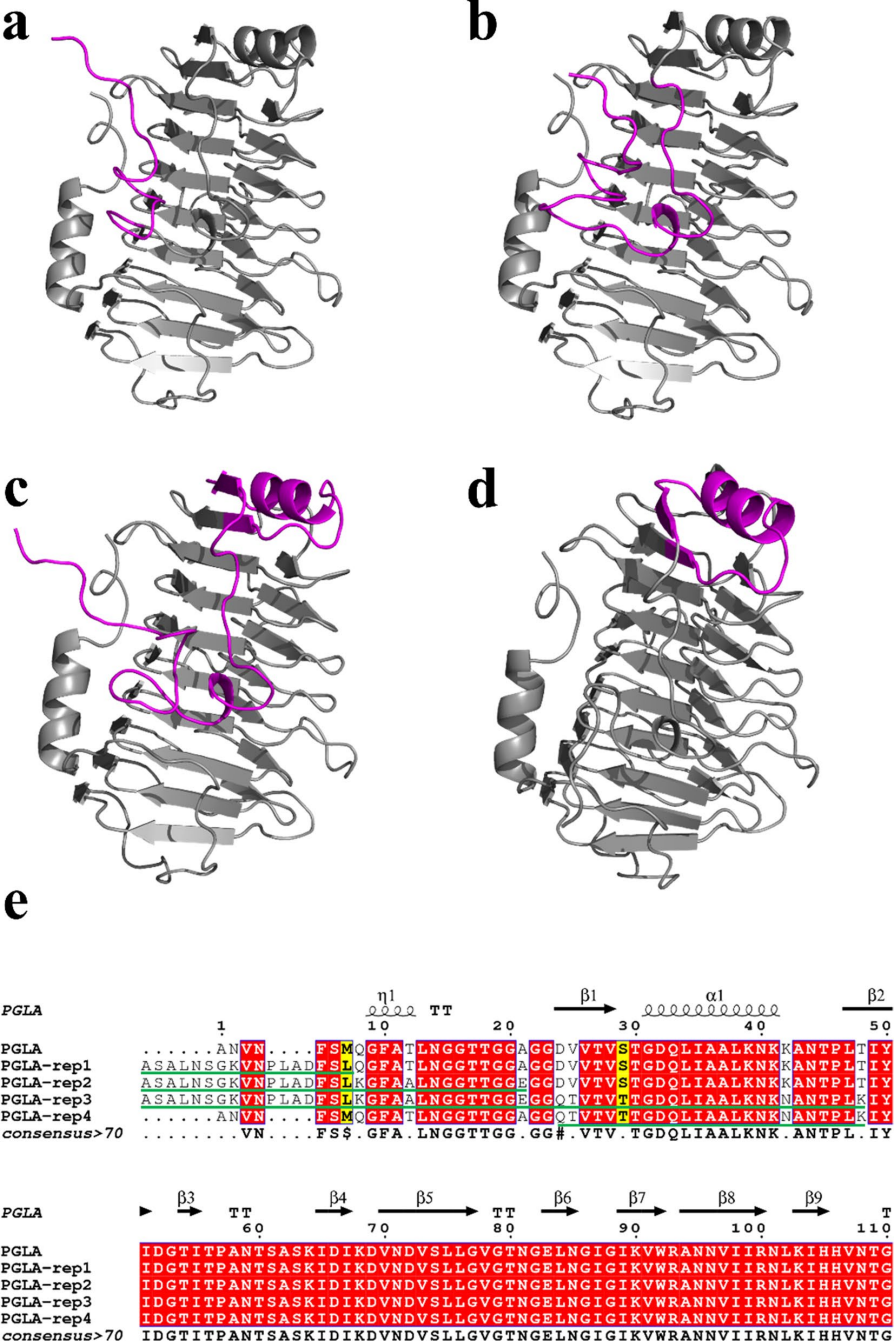
The three-dimensional structure and multiple sequence alignment of the modified alkaline pectin lyase **a** PGLA-rep1, **b** PGLA-rep2, **c** PGLA-rep3, **d** PGLA-rep4, **e** Constructed 5 sequence alignment maps, The part drawn with a green horizontal line is the part of the replacement fragment—the purple part in the 3D structure depicts replacement fragment

A signal peptide prediction identified the first 23 AAs of BacPelA from *B. clausii* as signal peptides (Zhou et al. 2017a). The recombinant PGLA, synthesized after removing the signal peptide, was used as the template. The fragments were replaced after sequence alignment with Pel SWU (Additional file 1: Fig. S2). The 3D structures of PGLA-rep1, PGLA-rep2, PGLA-rep3, and PGLA-rep4, after replacing the respective fragments, are viewed with PYMOL molecular visualization system (http://pymol.org/) (Fig. 1) PGLA-rep1, PGLA-rep2, and PGLA-rep3 had 17, 31, and 58 AAs replaced from the N-terminus, respectively, while PGLA-rep4 had 25 AAs replaced from Gln24 to Lys48. After the replacement of fragments, PGLA and PGLA-rep1-4 were produced by *E. coli* BL21(DE3), as described in “Materials and methods” section. The total activity of PGLA was 489.5 U/mL, while the enzyme activity of PGLA-rep4 was the highest at 554.0 U/mL. The enzymatic activities of PGLA-rep1, 2, and 3 were 413.8, 434.4, and 433.0 U/mL, respectively.

After purification by a nickel column, PGLA-rep4 had a specific enzyme activity of 822.9 U/mg, higher than the 664.1 U/mg of PGLA before replacement. Table 1 shows the enzymatic activity and specific enzymatic activity of purified pectin lyase before and after fragment replacement. SDS-PAGE analysis showed that the molecular weights of the four alkaline pectin lyases, after fragment replacement, were approximately 35 kDa (Additional file 1: Fig. S3).

**Table 1:** Comparison of properties of other reported pectin lyases

Pectin lyase	pH	Temperature/(°C)	Enzyme activity^a^/(U/mL)	Specific activity/(U/mg)	Culture medium	Expression host	Source of bacteria	References
BspPel	10	80	310	127.9	YPD medium	*Pichia pastoris*	*Bacillus* sp. RN1	Zheng et al. (2020)
Pel4J4	8.5	55	204.4	1059.4	LB medium	*E. coli* BL21	*D.dadantii* DCE-01	Cheng et al. (2019)
BacPelA	10.5	70	490.2	675.5	TB medium	*E. coli* BL21	*B.clausii* S10	Zhou et al. (2017a)
PpPel9a	10	40		298.5	LB medium	*E. coli* BL21	*Paenibacillus Polymyxa* KF-1	Yuan et al. (2019)
BliPelA	11	70	85.2	320	Modified TB medium	*E. coli* BL21	*Bacillus licheniformis* 91	Zhou et al. (2017c)
recPel S6	10	60		49.6	LB medium	*E. coli* BL21	*Bacillus amyloliquefaciens* S6	Bekli et al. (2019)
PGLA-rep1	10.5	70	413.8	604.9	TB medium	*E. coli* BL21	*B.clausii* S10	This work
PGLA-rep2	10.5	70	434.4	680.9	TB medium	*E. coli* BL21	*B.clausii* S10	This work
PGLA-rep3	11	65	433.0	472.5	TB medium	*E. coli* BL21	*B.clausii* S10	This work
PGLA-rep4	11	70	554.0	822.9	TB medium	*E. coli* BL21	*B.clausii* S10	This work

^a^The activity was determined by the A235 method, and the average value of three experiments was expressed

### Biochemical properties of modified alkaline pectin lyase

**Fig. 2:**
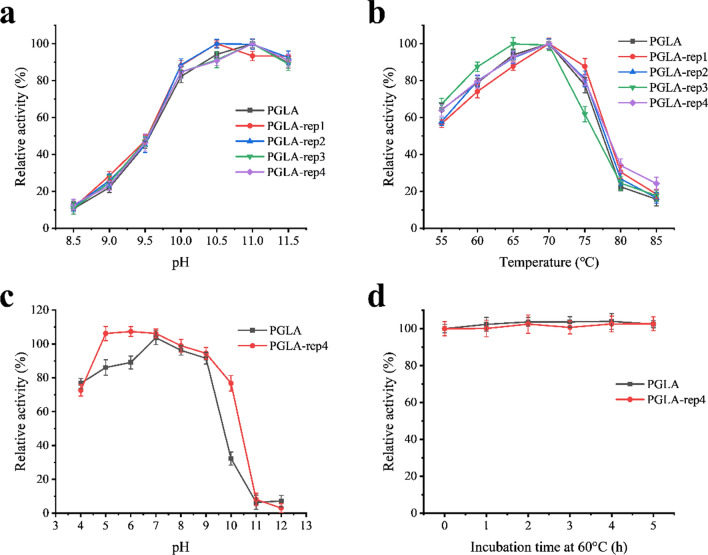
pH and temperature profiles of alkaline pectin lyase, **a** Optimum pH, **b** Optimum temperature, **c** Stability of PGLA and PGLA-rep4 at pH range, **d** Thermal stability of PGLA and PGLA-rep4 in glycine-NaOH buffer (pH 11.0) at 60 °C—the activities of all enzymes were determined under standard enzyme assay conditions using 0.2% apple pectin as substrate and all data are presented as the mean of three experiments; error bars represent standard deviation

The pH and temperature are two important evaluators of an enzyme’s properties. The pH of previously studied alkaline pectin lyases is mostly between 8.0 and 10.5, and the optimum temperature is generally between 50 and 65 °C (Chiliveri and Linga 2014; Shi et al. 2015; Wang et al. 2020; Zhou et al. 2017b). Using 0.2% pectin as a substrate, the optimum pH and temperature for PGLA, PGLA-rep1, PGLA-rep2, PGLA-rep3, and PGLA-rep4 were determined. The optimum pH of PGLA was 11.0, that of PGLA-rep1 and PGLA-rep2 was 10.5, and that of PGLA-rep3 and PGLA-rep4 was 11.0 (Fig. 2a). The modified lyase could still maintain > 40% activity at pH 9.5–11.5. The optimum temperature of PGLA, PGLA-rep1, PGLA-rep2, and PGLA-rep4 was 70 °C, while it was 65 °C for PGLA-rep3 (Fig. 2b). Alkaline pectin lyase maintained > 40% activity in the range of 55–75 °C. When the incubation temperature reached 85 °C, PGLA-rep4 maintained > 20% of its enzymatic activity.

The residual enzyme activities of PGLA and PGLA-rep4 after incubation at 50 °C for 7 h were determined to evaluate their pH stability (Fig. 2c). PGLA was stable in the range of pH 4–9 at 50 °C and maintained more than 70% of the enzyme activity. The results of PGLA-rep4 were identical to that of PGLA at pH 4–10. Additionally, the thermal stability of PGLA and PGLA-rep4 was determined after incubation at 60 °C at varying periods (Fig. 2d). After 5 h, the enzymatic activities of PGLA and PGLA-rep4 did not decrease, and the thermal stabilities before and after transformation were remarkably similar.

**Fig. 3:**
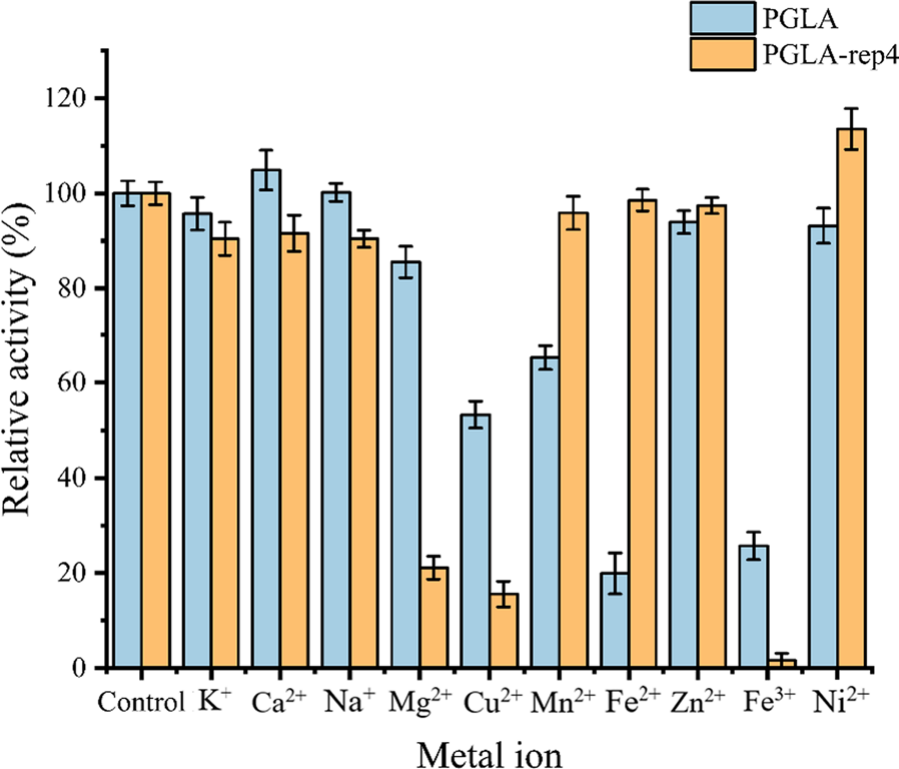
Effects of metal ions on the activity of PGLA and PGLA-rep4

The effect of metal ions on the activity of PGLA and PGLA-rep4 was evaluated at 25 °C (Fig. 3). Cu^2+^, Fe^2+^, and Fe^3+^ significantly reduced the activity of PGLA, whereas other metal ions had no significant effect. The activity of PGLA-rep4 was only significantly reduced in the presence of Mg^2+^, Cu^2+^, and Fe^3+^. Contrary to our results, previous studies have reported that Ca^2+^ activates the PGLA-rep4 and enhances its activity (Xu et al. 2021).

### Substrate spectroscopy and kinetic simulation of PGLA-rep4

**Fig. 4:**
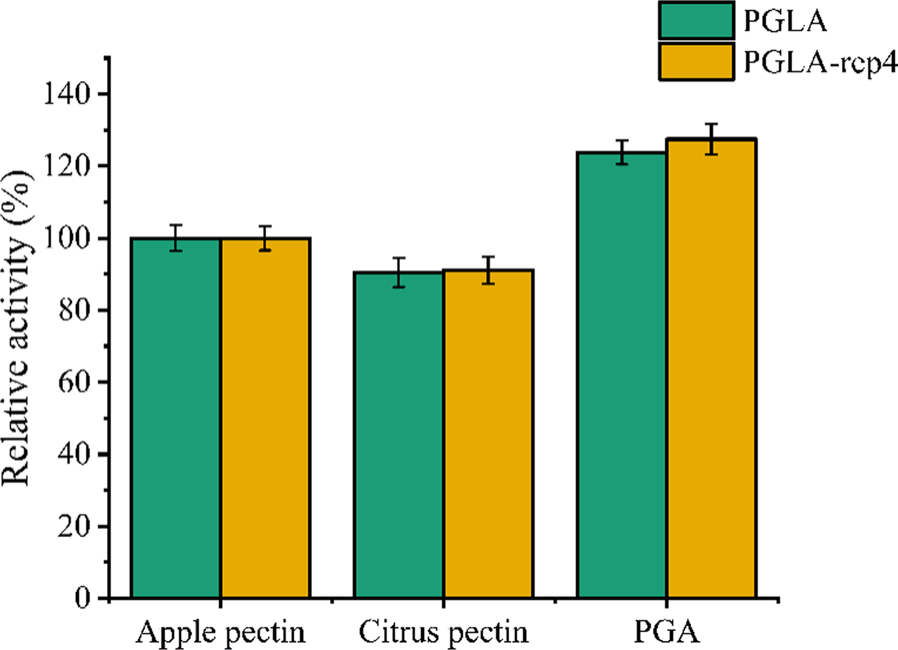
Substrate specificity of PGLA and PGLA-rep4

In terms of substrate specificity, PGLA and PGLA-rep4 showed high activity against apple and citrus pectin and PGA (Fig. 4). Taking the activity of PGLA and PGLA-rep4 on apple pectin as 100%, respectively, the activity of PGLA and PGLA-rep4 on citrus pectin were 90.5% and 91.0%, respectively, and the activity on PGA were 123.8% and 127.5%, respectively. All experimental data were repeated three times and averaged. Finally, we concluded that PGA was the preferred substrate for PGLA and PGLA-rep4.

**Fig. 5:**
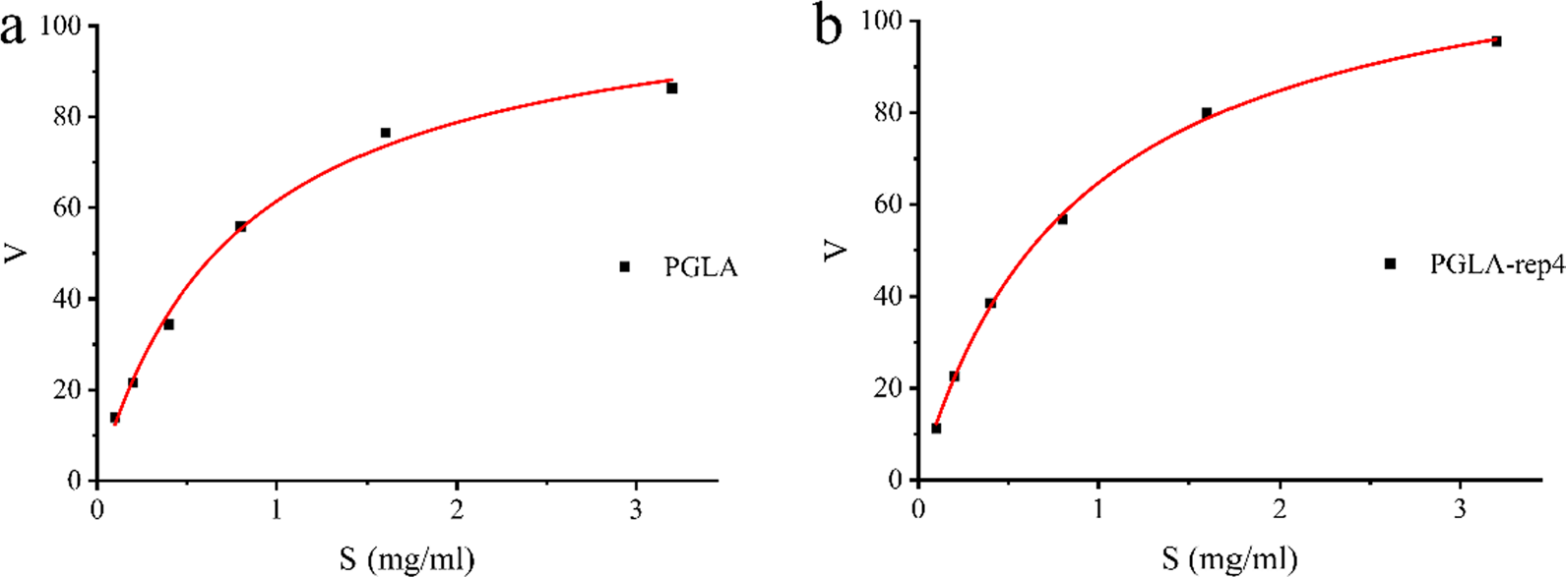
Nonlinear regression curves for the enzymatic reaction of pectin lyase. **a** PGLA reaction, **b** PGLA-rep4 reaction

Root-mean-square deviation (RMSD) was used to assess the extent to which the structure deviated from the initial configuration and explored the structural stability of pectin lyases. The RMSD values, relative to the initial structure, were plotted as a function of time during a 20 ns molecular dynamics simulation run (Additional file 1: Fig. S4). Under unconstrained conditions, PGLA and PGLA-rep1, PGLA-rep2, PGLA-rep3, and PGLA-rep4 reached equilibrium at approximately 10,000 ps, and after which the mean RMSD was 0.538, 0.551, 0.643, 0.555, and 0.634 nm, respectively. The similarity of these results is indicative of stable molecular dynamics after fragment replacement. Root mean square fluctuation (RMSF) was used to measure the average AA flexibility of PGLA and PGLA-rep4 from the initial frame (Additional file 1: Fig. S5). PGLA fluctuated to a greater degree and had a higher RMSF value. This may explain the higher enzymatic activity of PGLA-rep4 after fragment replacement. Conversely, the K_m_ and V_max_ values of PGLA and PGLA-rep4 were calculated using a nonlinear regression method (Fig. 5). The K_m_ and V_max_ values of PGLA were 0.79 g/L and 109.81 nkat/mg protein, respectively. The K_m_ and V_max_ values of PGLA-rep4 were 0.90 g/L and 123.06 nkat/mg protein, respectively. In conclusion, although the affinity of pectin lyase PGLA-rep4 for the substrate was not as strong as that of PGLA, its catalytic ability was much higher than that of PGLA.

## Discussion

Zhou et al. (2017a) discovered the alkaline pectate lyase BacPelA (accession no. KR819891.1). This enzyme has an optimum temperature and pH of 70 °C and 10.5, respectively, and, to date, the highest reported expression activity was 8378.2 U/mL after high-density fed-batch culture and fermentation. In this study, we replaced the unstable region of the N-terminus of pectin lyase PGLA with four fragments after aligning PGLA with Pel SWU (Additional file 1: Fig. S6). Replacement fragment 4 was based on replacement fragment 3. The harmful components of replacement fragments 1 and 2 were removed. The enzymatic properties of each modified enzyme were evaluated. Despite the unaffected physicochemical characteristics of PGLA-rep4, its enzymatic activity was significantly improved. Compared with PGLA, the pH and thermal stability of PGLA-rep4 did not change significantly. Thus, we can conclude that N-terminal fragment replacement can significantly improve the enzymatic activity of pectin lyase without altering its enzymatic properties. This feature differs from that reported in previous studies. You et al. (2019) found that the thermostability of GH10 xylanase XyIE increased at temperatures from 70 to 75 °C after fragment replacement. In this study, however, fragment replacement did not improve the thermostability of PGLA but rather its enzymatic activity. The enzymatic activity of PGLA-rep4 was higher than that of the other four previously reported bacterial pectin lyases (Table 1): the pectin lyase pel4J4 with a total activity of 204.4 U/mL (Cheng et al. 2019), the pectin lyase reported by Yuan et al. (2019) with a specific enzyme activity of 298.5 U/mg, the pectate lyase studied by Zhou et al. (2017b) with a total activity of 85.2 U/mL after 24 h incubation in shake flasks with TB medium and a specific activity after purification of 320 U/mg, and the pectin lyase studied by Bekli et al. (2019) with the specific enzyme activity of 49.6 U/mg after purification. In addition, the relative activity of PGLA-rep4 in the range of pH 4–10 was still more than 70% after being stored at 50 °C for 7 h, indicating good pH stability. The specific enzyme activity of PGLA-rep4 purified by a nickel column was 374.1 U/mg. Based on the specific enzyme activity, the enzyme concentration in the PGLA-rep4 fermentation broth could be converted to 1.1 mg/mL.

**Fig. 6:**
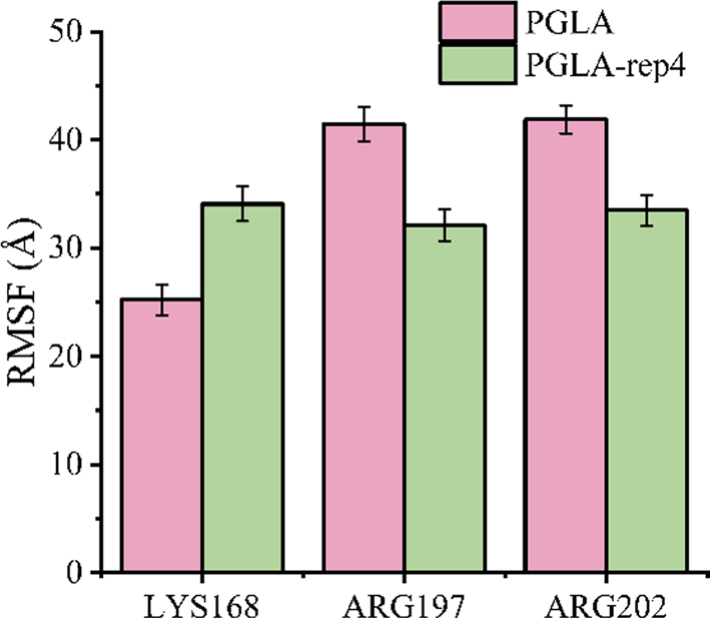
RMSF values of PGLA and PGLA-rep4 at three key catalytic sites

In recent years, research on alkaline pectin lyases has been increasing. However, there are very few reported pectin lyases with an optimum temperature of 70 °C and an optimum pH of 11.0. Considering that PGLA-rep4 has these optimal characteristics, in addition to its high catalytic activity for apple pectin, citrus pectin, and PGA, and its relatively stable structure, it can be applied to ramie, cotton, and other degumming processes. The three key AAs at the catalytic site of pectate lyase are LYS168, ARG197, and ARG202 (Zhou et al. 2017a); the RMSF values of PGLA and PGLA-rep4 at these three active sites were shown in Fig. 6. It can be seen from the figure that LYS168 was the key AA for the cleavage substrate; therefore, the catalytic activity of PGLA-rep4 was higher than that of PGLA. PGLA-rep4 has great potential for degumming by enzymatic-chemical combined processes in textile processing, pulp and paper, feed processing, and other industries and provides the possibility of innovative development in other industries.

In conclusion, this study provides a strategy for enhancing alkaline pectin lyase activity. The modified alkaline pectin lyase PGLA-rep4 in this study had high alkali resistance and good thermal stability, especially for the substrates apple pectin, citrus pectin, and PGA. It is relatively structurally stable, can be used over a wide pH range, and is stable in the presence of metal ions. These findings indicate that PGLA-rep4 has broad application potential in the textile, pulp and paper, and food industries and can be of huge commercial value.

## Supplementary Information

**Additional file 1: Table S1.** Oligonucleotides used in this study. **Fig. S1.** Nucleotide sequence alignment of codon-unoptimized PGLA and codon-optimized PGLA. **Fig. S2.** SDS-PAGE plot during fragment replacement. a lane M, markers; lane 1, 2, 3, heat-resistant fragment rep2; lane 5, 6, 7, heat-resistant fragment rep3. b lane M, markers; lane 1, 2, 3, heat-resistant fragment rep4. c lane M, markers; lane 1, 2, linearized vector 2; lane 3, 4, linearized vector 3; lane 5, 6, linearized vector 4. d lane M, markers; lane 1, 2, 3, 4, the recombinant plasmid pET28a-PGLA-rep1 was amplified by inverse PCR. **Fig. S3.** SDS-PAGE Analysis of pectin lyase with the lanes showing the varying contents of supernatant of crude extract of *E. coli* BL21(DE3). Lane 1, pET28a(+)-*PGLA*; Lane 2, pET28a(+)-*PGLA-rep1*; Lane 3, pET28a(+)-*PGLA-rep2*; Lane 4, pET28a(+)-*PGLA-rep3*; Lane 5, pET28a(+)-*PGLA-rep4*; Lane M, molecular weight markers. **Fig. S4.** Molecular dynamics simulation of the RMSD value curves of PLGA, PGLA-rep1, PGLA-rep2, PGLA-rep3 and PGLA-rep4 for 20 ns. **Fig. S5.** RMSF value curves of PGLA and PGLA-rep4. **Fig. S6.** Amino acid sequence alignment of PGLA and Pel SWU.

## Data Availability

All experimental data generated in this study are available on request to the corresponding authors if required.
