# Supplementary material for: Modification and application of highly active alkaline pectin lyase
Source: AMB Express. 2022 Oct 9;12:130. doi: 10.1186/s13568-022-01472-0 (PMC9548460; doi:10.1186/s13568-022-01472-0)
Supplement: Supplementary file 1 — Additional file 1: Table S1. Oligonucleotides used in this study. Fig. S1. Nucleotide sequence alignment of codon-unoptimized PGLA and codon-optimized PGLA. Fig. S2. SDS-PAGE plot during fragment replacement. a lane M, markers; lane 1, 2, 3, heat-resistant fragment rep2; lane 5, 6, 7, heat-resistant fragment rep3. b lane M, markers; lane 1, 2, 3, heat-resistant fragment rep4. c lane M, markers; lane 1, 2, linearized vector 2; lane 3, 4, linearized vector 3; lane 5, 6, linearized vector 4. d lane M, markers; lane 1, 2, 3, 4, the recombinant plasmid pET28a-PGLA-rep1 was amplified by inverse PCR. Fig. S3. SDS-PAGE Analysis of pectin lyase with the lanes showing the varying contents of supernatant of crude extract of E. coli BL21(DE3). Lane 1, pET28a(+)-PGLA; Lane 2, pET28a(+)-PGLA-rep1; Lane 3, pET28a(+)-PGLA-rep2; Lane 4, pET28a(+)-PGLA-rep3; Lane 5, pET28a(+)-PGLA-rep4; Lane M, molecular weight markers. Fig. S4. Molecular dynamics simulation of the RMSD value curves of PLGA, PGLA-rep1, PGLA-rep2, PGLA-rep3 and PGLA-rep4 for 20 ns. Fig. S5. RMSF value curves of PGLA and PGLA-rep4. Fig. S6. Amino acid sequence alignment of PGLA and Pel SWU. [file 13568_2022_1472_MOESM1_ESM.docx]

**AMB Express**

**Modification and application of highly active alkaline pectin lyase**

Pi-Wu Li^1,2#^, Jun Ma^2#^, Xiao-Feng Wei^2^, Zi-Yang Zhang^2^, Rui-Ming Wang^1,2^, Jing Xiao^1,2^, Jun-Qing Wang^1,2*^

1 State Key Laboratory of Biobased Material and Green Papermaking (LBMP) (Qilu University of Technology), Jinan 250353, Shandong, Republic of China.

2 Key Laboratory of Shandong Microbial Engineering, Qilu University of Technology (Shandong Academy of Sciences), Jinan 250353, Shandong, Republic of China.

#Piwu Li and Jun Ma are co-first authors of the article.

*Author for correspondence: Junqing Wang

Tel: +86-0531-89631138

Fax: +86-0531-89631138

E-mail: wjqtt.6082@163.com

**Table S1 Oligonucleotides used in this study**

| Primers | Sequences | Sources |
| --- | --- | --- |
| PGLA1-F | CGGGCAAAGTAAATCCGCTTGCCGACTTCAGCTTACAAGGTTTTGCCACTCTCAATG | This work |
| PGLA1-R | AAGCGGATTTACTTTGCCCGAGTTTAAGGCAGAAGCCATGGTATATCTCCTTCTTAAAGTTAAAC | This work |
| PGLA2-F | GGGGGTGATGTGGTGACCG | This work |
| PGLA34-F | ATCTATATCGATGGTACCATCACCC | This work |
| PGLA23-R | CATGGTATATCTCCTTCTTAAAGTTAAACA | This work |
| PGLA4-R | ACCCCCCGCACCGCCTGT | This work |
| rep23-F | taagaaggagatataccatgGCTTCTGCCTTAAACTCGGGC | This work |
| rep2-R | acggtcaccacatcacccccTTCTCCGCCCGTTGTTCC | This work |
| rep3-R | taagaaggagatataccatgGCTTCTGCCTTAAACTCGGGC | This work |
| rep4-F | ccacaggcggtgcggggggtCAGACGGTAACCGTAACAACGG | This work |
| rep4-R | atggtaccatcgatatagatTTTTAAAGGCGTATTTGCATTCTT | This work |

Restriction endonuclease digestion sites were underlined.

**
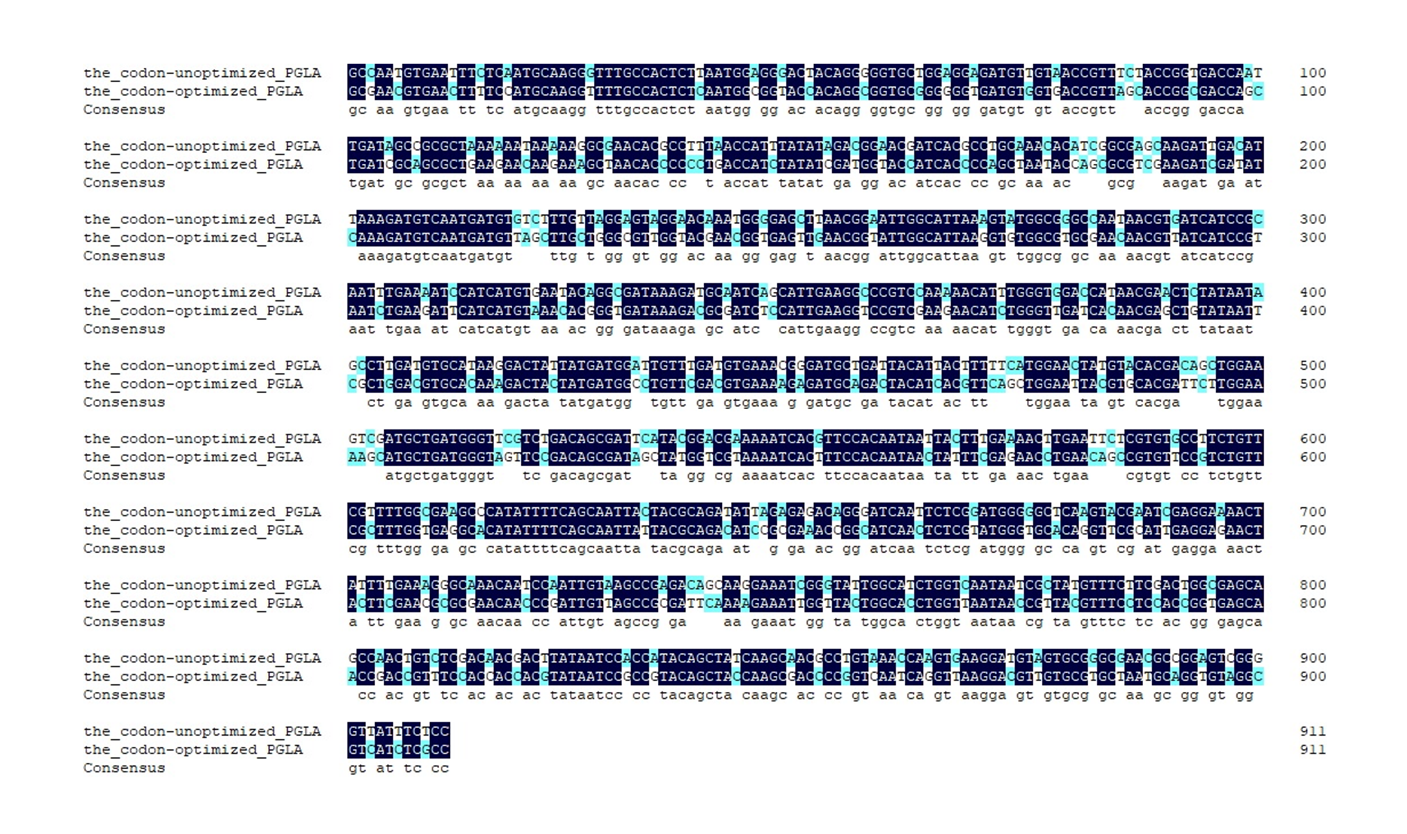
Fig. S1 Nucleotide sequence alignment of codon-unoptimized PGLA and codon-optimized PGLA**

**
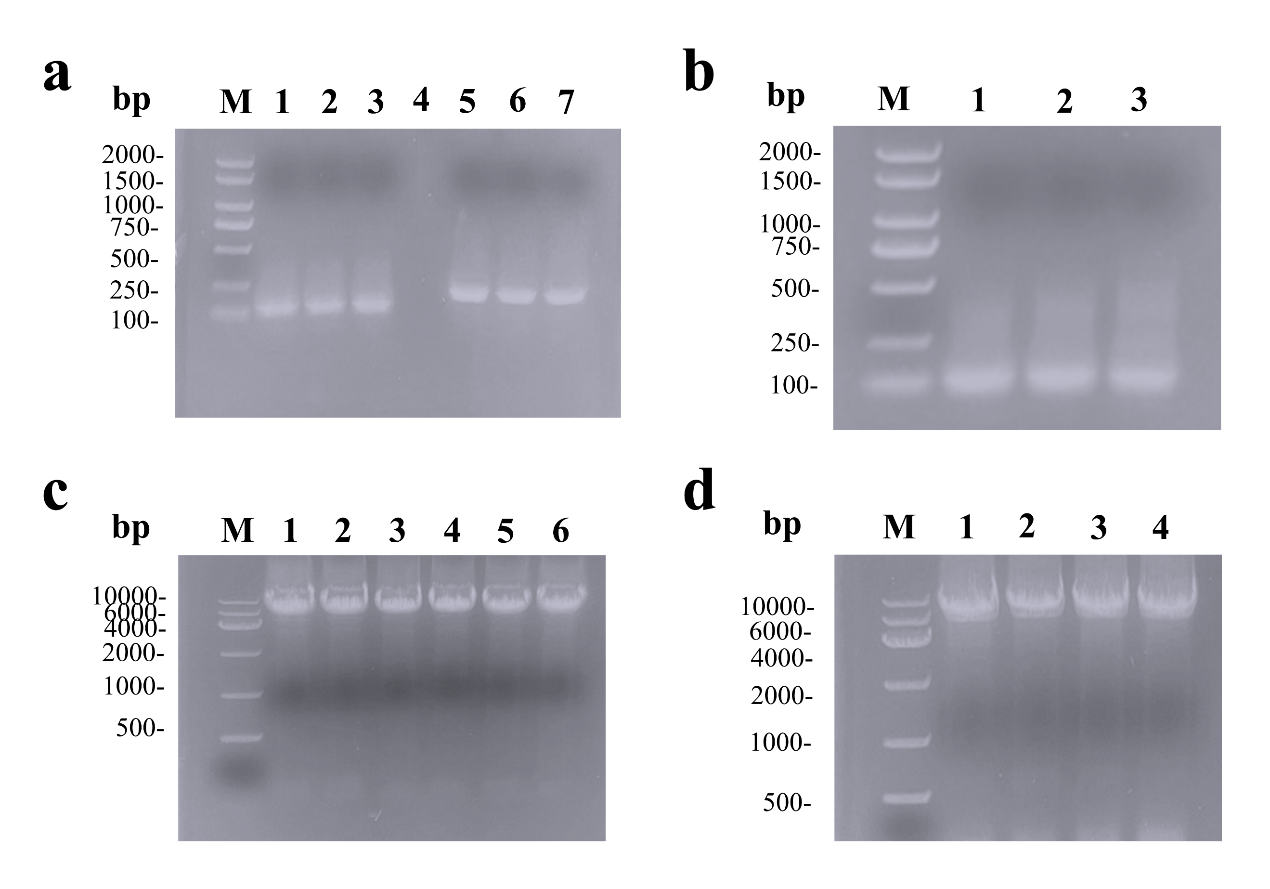
**

**Fig. S2 SDS-PAGE plot during fragment replacement.** a lane M, markers; lane 1, 2, 3, heat-resistant fragment rep2; lane 5, 6, 7, heat-resistant fragment rep3. b lane M, markers; lane 1, 2, 3, heat-resistant fragment rep4. c lane M, markers; lane 1, 2, linearized vector 2; lane 3, 4, linearized vector 3; lane 5, 6, linearized vector 4. d lane M, markers; lane 1, 2, 3, 4, the recombinant plasmid pET28a-PGLA-rep1 was amplified by inverse PCR


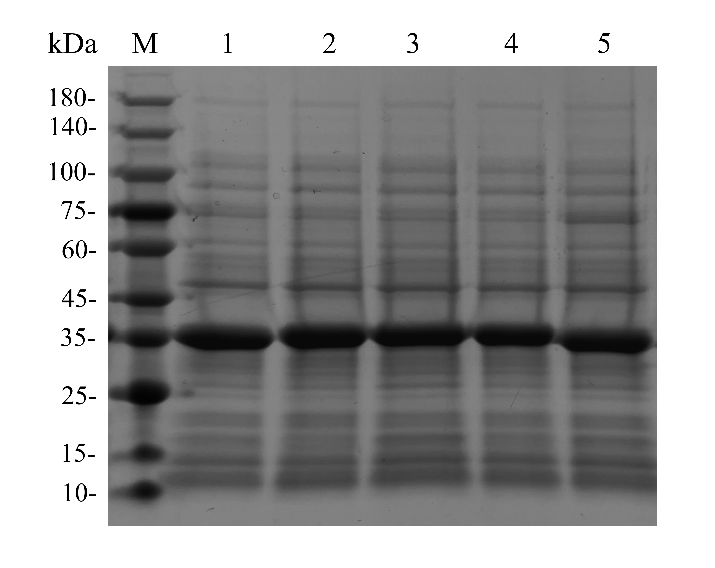


**Fig. S3 SDS-PAGE Analysis of pectin lyase with the lanes showing the varying contents of supernatant of crude extract of *E.coli* BL21(DE3).** Lane 1, pET28a(+)-*PGLA*; Lane 2, pET28a(+)-*PGLA-rep1*; Lane 3, pET28a(+)-*PGLA-rep2*; Lane 4, pET28a(+)-*PGLA-rep3*; Lane 5, pET28a(+)-*PGLA-rep4*; Lane M, molecular weight markers


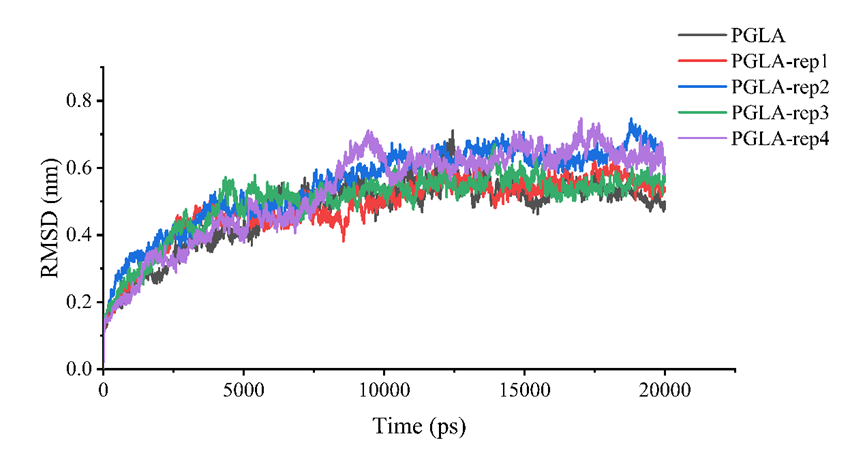


**Fig. S4 Molecular dynamics simulation of the RMSD value curves of PLGA, PGLA-rep1, PGLA-rep2, PGLA-rep3 and PGLA-rep4 for 20 ns**

**
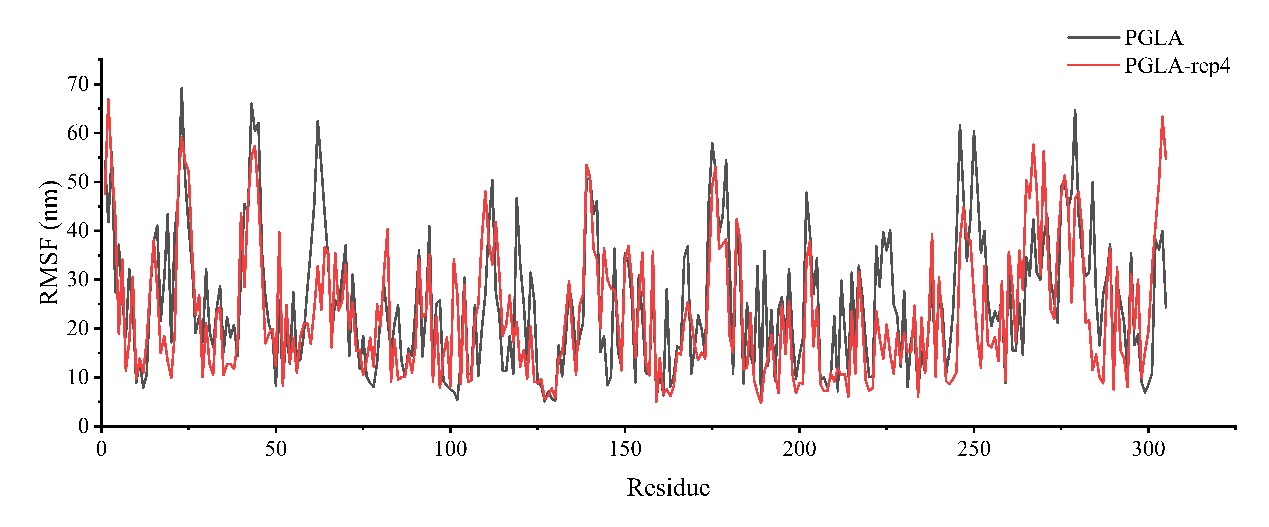
**

**Fig. S5 RMSF value curves of PGLA and PGLA-rep4**

**
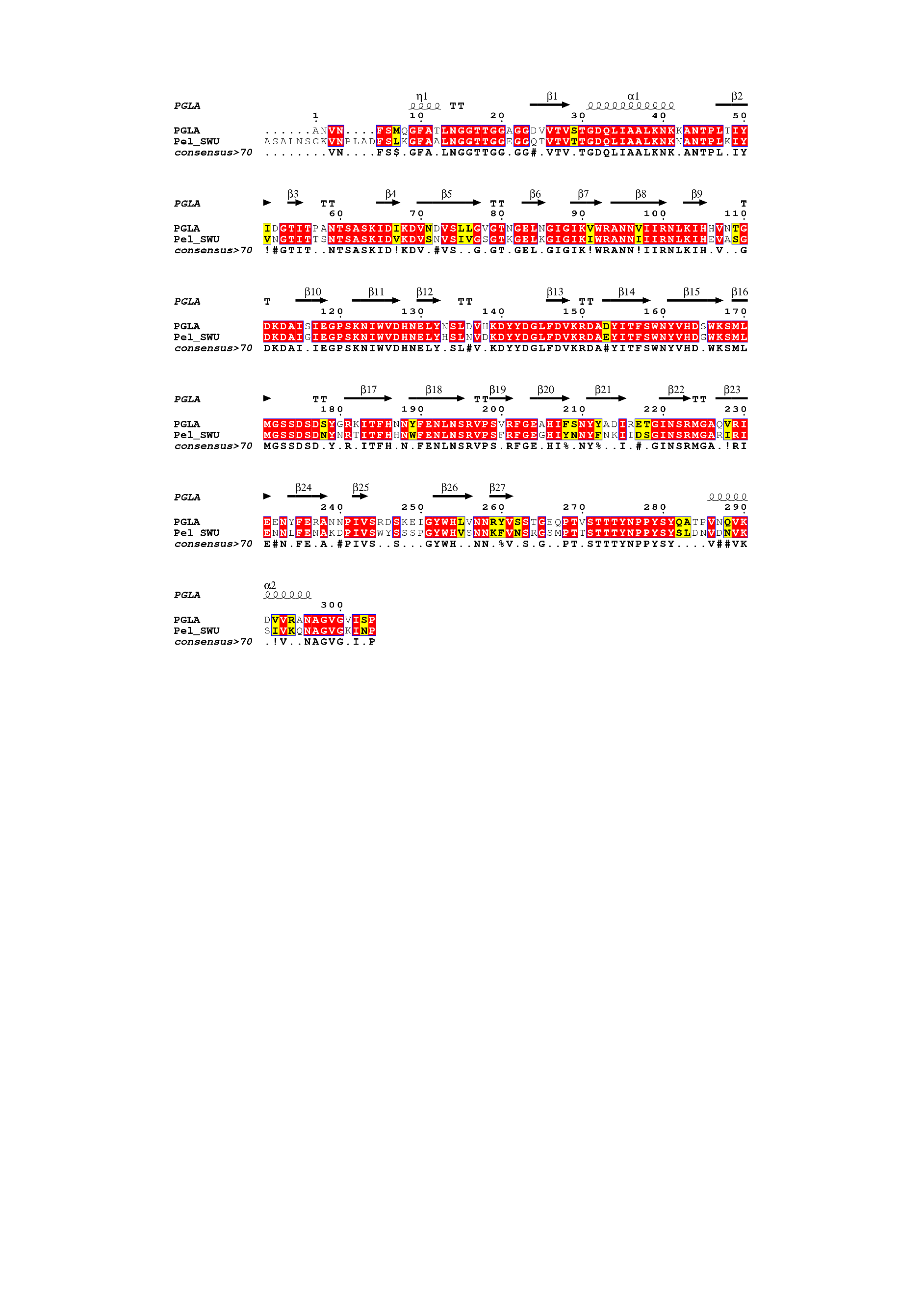
**

**Fig. S6 Amino acid sequence alignment of PGLA and Pel SWU**
